# YOLO-WildASM: An Object Detection Algorithm for Protected Wildlife

**DOI:** 10.3390/ani15182699

**Published:** 2025-09-15

**Authors:** Yutong Zhu, Yixuan Zhao, Yanxin He, Baoguo Wu, Xiaohui Su

**Affiliations:** 1 School of Information Science and Technology, Beijing Forestry University, Beijing 100083, China; zhuyutong@bjfu.edu.cn (Y.Z.); zyxuan@bjfu.edu.cn (Y.Z.); hyx036900@bjfu.edu.cn (Y.H.); wubg@bjfu.edu.cn (B.W.); 2 Engineering Research Center for Forestry-Oriented Intelligent Information Processing, National Forestry and Grassland Administration, Beijing 100083, China; 3 Key Laboratory of Smart National Park in Hebei Province, Beijing 100083, China; 4 Research Institute of Forestry Informatization, Beijing Forestry University, Beijing 100083, China

**Keywords:** wildlife, deep learning, object detection, You Only Look Once, small object detection, adaptive multi-scale fusion, attention mechanism

## Abstract

Wild animals are an essential component of natural ecosystems, and the accurate identification of wildlife targets plays a critical role in ecological conservation and species monitoring. However, the effectiveness of conventional object detection algorithms is often limited by the challenges posed by complex outdoor environments, small target sizes, and group occlusions. To address these issues, this study constructs a dataset comprising over 8000 images of 10 protected wildlife species and investigates effective detection methods for wildlife in natural habitats. We propose a novel deep learning-based detection framework, YOLO-WildASM, which incorporates three key improvements to the YOLOv8 architecture: a P2 detection layer for small objects, a multi-head self-attention (MHSA) mechanism, and a bidirectional feature pyramid network (BiFPN). Experimental results demonstrate that YOLO-WildASM significantly outperforms YOLOv8 and other state-of-the-art models on the custom wildlife dataset, achieving a mAP50 of 94.1%, which is 2.8% higher than the baseline model and superior to the latest YOLOv12 model (92.2%). Furthermore, ablation and generalization experiments validate the model’s enhanced performance and adaptability in multi-scale wildlife detection tasks. The proposed deep learning-based detection framework provides an efficient and robust solution for wildlife monitoring and ecological conservation in complex natural ecosystems.

## Simple Summary

Effective monitoring and object detection of wildlife in complex natural environments is crucial for species conservation, and object detection technology serves as a key component of modern monitoring systems. This study addresses challenges such as small object detection, occlusions, and the coexistence of multiple targets in complex backgrounds by proposing an enhanced model, YOLO-WildASM, based on YOLOv8. The proposed model incorporates a P2 detection layer for small objects, BiFPN-based feature fusion, and a multi-head self-attention (MHSA) mechanism to significantly improve detection accuracy. Experimental results demonstrate that YOLO-WildASM achieves a mAP50 of 94.1%, representing a 2.8% improvement over YOLOv8. These results indicate that YOLO-WildASM effectively meets the requirements for fast and accurate wildlife identification in the wild, offering valuable technical support for ecological protection and species monitoring.

## 1. Introduction

Recent years have witnessed escalating human disturbances, including habitat loss, illegal hunting, and changes in predator–prey dynamics, which have become major drivers of biodiversity loss and have reshaped wildlife distribution and behavior across ecosystems [1,2,3,4]. Scientific and effective monitoring forms the foundation of wildlife conservation. Rapid and accurate monitoring of wildlife species, including their population dynamics, habitat conditions, and behavioral patterns, is essential both for understanding the impacts of threats such as habitat loss, hunting, and climate change, and for supporting the development of effective conservation policies [5,6]. Traditional wildlife monitoring methods—such as mark-recapture, expert surveys, and radio telemetry collars [7,8]—though demonstrating certain effectiveness, suffer from high costs, low efficiency, constraints from weather and geographic conditions, and potential disturbances to wildlife behavior and ecosystems. These limitations hinder their ability to meet current biodiversity conservation demands [9,10,11,12].

With the advancement of modern information technologies, infrared cameras and acoustic devices have become vital sources of biodiversity big data [13,14]. Infrared cameras capture changes in infrared radiation triggered by animal body heat and characteristics, enabling the recording of animal behaviors, population densities, and distributions. They are particularly suited for monitoring nocturnal species and provide efficient, reliable image data without disturbing wildlife activities [15,16,17]. Concurrently, with the continuous development of machine learning technologies, deep learning-based object detection techniques have matured significantly. These techniques enable automated and efficient identification of wildlife images obtained from monitoring devices, accurately tracking behaviors, population sizes, and distribution patterns. This has emerged as a revolutionary technology in wildlife conservation [18,19,20]. Among these, the YOLO (You Only Look Once) series of algorithms [21] holds a prominent position in the object detection field due to its high real-time efficiency and robust detection accuracy. Compared to traditional region proposal network (RPN) methods, the YOLO algorithm employs a single neural network to perform object detection and classification directly on the entire image, significantly reducing computational complexity. It maintains high accuracy while enabling rapid detection, making it widely applicable to real-time object detection tasks across diverse scenarios. YOLO stands as a pivotal technology in modern object detection.

In recent years, the rapid development of the YOLO series in object detection algorithms has established it as a core technology for wildlife conservation and monitoring, widely applied in image and video data detection and analysis. Numerous scholars have proposed innovative methods by optimizing YOLO models to address challenges posed by complex field environments and wildlife characteristics. For instance, Zhang et al. [22] incorporated a lightweight GSConv module into YOLOv5s and replaced the C3 module with a GS bottleneck structure, effectively alleviating the low recognition accuracy for large animals in complex environments. Ma et al. [23] enhanced the detection precision and inference speed of YOLOv5 for wildlife by integrating a MobileNetV3 feature extraction module with a CBAM attention mechanism. Su et al. [24] further optimized the C3 module in YOLOv5 by introducing a NAMAttention mechanism and a MemoryEfficientMish activation function, significantly suppressing interference from irrelevant features and low-quality images. Chen et al. [25] enhanced YOLOv8n by introducing the Softplus activation function and by constructing an AIFI-Slim feature fusion structure, significantly suppressing background interference and misdetection phenomenon in wildlife monitoring while effectively balancing detection accuracy and real-time requirements. Furthermore, to address wildlife occlusion challenges, He [26] proposed an Adaptive Lightweight Channel Split and Shuffle (ALSS) module, which optimizes blurred feature extraction and mitigates issues caused by motion blur and object overlap. For small-target detection, Mou et al. [27] introduced a dedicated small-object module into the YOLOv7 detection head, resolving detection difficulties for tiny targets in drone imagery. Jiang et al. [28] enhanced small-object detection performance in complex backgrounds by integrating an EMGA attention mechanism and an ASPFC feature fusion module. Roy et al. [29] optimized the backbone network by integrating DenseNet-based dense feature extraction modules with dual residual blocks, and fused an SPF-PANet multi-scale feature fusion structure, significantly suppressing interference from complex environments and localization deviations in low-quality samples. He et al. [26] further refined localization accuracy by adjusting the localization loss function to emphasize weights associated with small targets. Despite these advancements, wildlife object detection continues to face accuracy challenges due to cluttered backgrounds, variable lighting conditions, significant scale variations among wildlife targets, and the unique characteristics of animal group behaviors in natural environments [30].

To address the challenges of detecting wildlife in complex field environments characterized by ultra-small targets, mutual occlusion, and multi-target coexistence, this study proposes YOLO-WildASM, an improved YOLOv8-based model developed through three key enhancements: introducing a P2 ultra-small target detection layer, integrating BiFPN (Bidirectional Feature Pyramid Network) for advanced feature fusion, and incorporating MHSA (Multi-Head Self-Attention) mechanisms. Leveraging a self-constructed dataset, we systematically validate the model’s effectiveness, superior performance, and extensibility through comprehensive ablation studies, comparative experiments, and cross-dataset generalization tests. The principal contributions of this research are as follows:

1. We established a specialized wildlife dataset comprising over 8000 images of 10 protected species (giant panda, leopard, tiger, snow leopard, wolf, red fox, black bear, red panda, yellow-throated marten, and otter) collected through verified channels. Video frame differencing techniques were applied to expand image diversity from collected video sources. All data were annotated via LabelImg 1.8.6, ensuring comprehensive coverage of complex backgrounds, diverse postures, multi-target/small-target scenarios, and diurnal/nocturnal conditions.
2. The proposed model enhances small-object detection by incorporating a P2/4 layer (160 × 160 resolution) into YOLOv8′s PAN-FPN neck network. This modification preserves high-resolution spatial details for targets smaller than 32 × 32 pixels. By propagating detailed shallow features to deeper network layers, the optimization mitigates conventional FPN limitations while improving spatial localization for larger objects through enriched feature representation.
  We replace the original concatenation layers with BiFPN (Bidirectional Feature Pyramid Network)-based weighted fusion modules, including dual-channel BiFPN_Concat2 and triple-channel BiFPN_Concat3. This architecture strengthens the model’s capacity to concurrently detect targets for multi-target coexistence in complex environments.
3. YOLO-WildASM incorporates Multi-Head Self-Attention (MHSA) mechanisms to address occlusion challenges and complex background interference. The parallel computation of multiple attention heads captures global contextual dependencies, enhancing feature discriminability for occluded targets and cluttered environments. Synergizing with the P2 layer and BiFPN enhancements, this integration substantially promotes detection accuracy in multi-object scenarios, providing robust technical support for wildlife monitoring applications.

## 2. Materials and Methods

### 2.1. Data and Preprocessing

This study systematically selected 10 protected species (leopard, giant panda, tiger, snow leopard, red fox, yellow-throated marten, wolf, black bear, otter, and red panda) as research subjects, considering their diverse body sizes and habitat requirements across complex ecosystems, including forests, grasslands, aquatic environments, deserts, and mountainous regions. Through collaboration with the Chengdu Institute of Biology, iNaturalist [31], the Land-Bridge Ecological Center (EBC), and the China Conservation and Research Center for the Giant Panda, we collected 8174 validated images documenting multiple behavioral postures of these species (Table 1). Some image data were extracted from video frames. Based on changes in animal movement speed in the videos, frames were captured at a variable rate—ranging from 3 frames per second to 1 frame every 5 s—using significant changes in animal posture as the extraction criterion. This approach expanded the image dataset. The dataset specifically emphasizes inter-occlusion scenarios and intraspecific postural variations under challenging conditions, including illumination changes, background clutter, and scale variations. All images were captured using field-deployed infrared cameras and conventional photographic equipment, ensuring data diversity and ecological representativeness for robust wildlife monitoring applications.

Following image acquisition, annotation was performed using the open-source tool LabelImg. This tool supports output in both YOLO (.txt) and Pascal VOC (.xml) formats and is widely employed for image annotation and dataset preparation. Specific emphasis was placed on ensuring the completeness and consistency of wildlife target annotations during this process. Completeness necessitates that every target within an image is fully annotated, with bounding boxes tightly enclosing the target edges. Consistency requires that targets belonging to the same category conform to uniform annotation criteria, ensuring boundary box delineation standards are maintained without arbitrary alterations. Then, the dataset was partitioned into training, validation, and test sets in a 6:2:2 ratio using a custom Python 3.8 script. This resulted in 4906 images for training, 1634 for validation, and 1634 for testing, with corresponding annotation files systematically allocated to each subset.

### 2.2. The YOLO-WildASM Model

**Figure 1:**
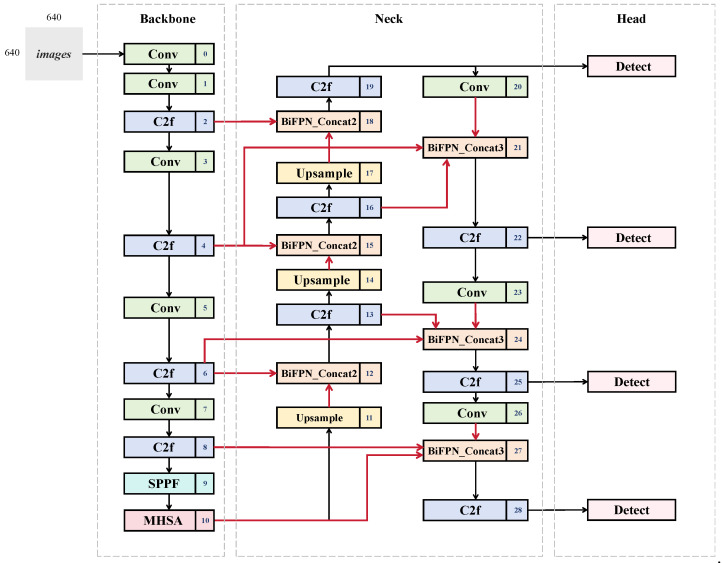
Illustration of YOLO-WildASM Model Architecture.

The YOLO-WildASM model was established based on YOLOv8, the single-state detection model. To improve the efficiency and accuracy of detecting small objects, multiple coexisting targets, occlusions, and recognizing objects in a complex environment, the improved model integrates P2 Feature Pyramid Layer and Multi-Head Self-Attention mechanism, and introduces the BiFPN_Concat-Driven Feature. The term “ASM” represents the tree core improvements integrated into the model: Attention Mechanism, Small Object Detection Layer, and Multi-Scale Feature Fusion. The framework is shown in Figure 1. In the figure, the red arrows highlight the newly introduced feature fusion and upsampling paths, which represent the key improvements of our YOLO-WildASM design. The YOLO-WildASM model incorporates all three strategies into a 28-layer architecture with four detection heads, which ensures robust recognition in complex scenarios while avoiding excessive computational overhead associated with overly deep networks.

#### 2.2.1. YOLOv8

YOLOv8 [32], released by Ultralytics in January 2023, preserves the single-stage detection paradigm of the YOLO series while adopting an end-to-end architecture to directly predict object categories and bounding box coordinates from input images, making it particularly suitable for real-time applications such as wildlife monitoring and autonomous driving.

**Figure 2:**
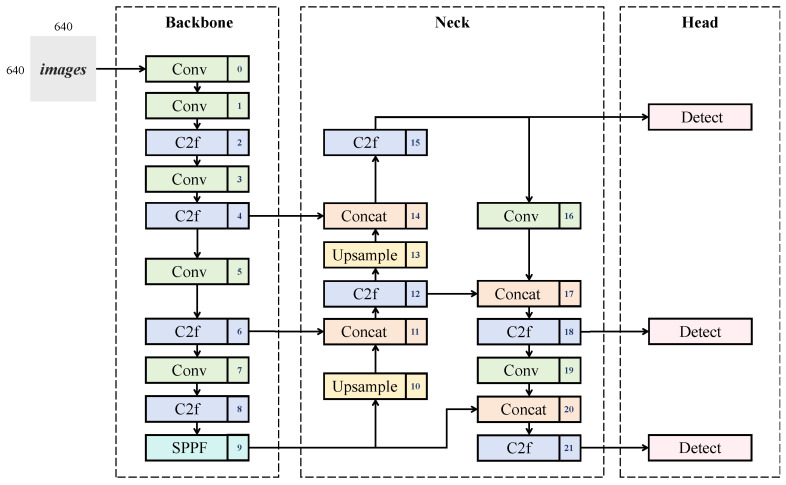
The overall structure of the YOLOv8 model.

The architecture of YOLOv8 comprises three core components: the backbone, neck, and head networks (Figure 2). The backbone network employs a hierarchical downsampling mechanism to generate multi-scale feature maps, extracting rich semantic information through an enhanced CSP-Darknet (Cross Stage Partial Darknet) structure [32] integrated with YOLOv7’s ELAN design [33]. By replacing YOLOv5’s C3 module with a novel C2f module, YOLOv8 optimizes gradient flow propagation, thereby improving feature extraction efficiency and achieving model lightweighting. The neck network enhances multi-scale object detection capability through feature fusion, utilizing a streamlined Path Aggregation Feature Pyramid Network (PAN-FPN) [34] inherited from YOLOv5. This architecture eliminates redundant 1 × 1 downsampling layers while maintaining bidirectional feature aggregation paths (deep-to-shallow and shallow-to-deep) to refine multi-scale integration. The head network adopts a decoupled design that separates classification and bounding box regression tasks, coupled with an anchor-free mechanism for direct regression of target center points and dimensions. This approach reduces hyperparameter dependency and improves generalization compared to traditional coupled architectures. The optimized loss function combines Binary Cross-Entropy (BCE) for classification, Distribution Focal Loss (DFL) for positional distribution modeling, and Complete Intersection over Union (CIoU) loss for geometric consistency, replacing conventional confidence loss to enhance regression accuracy. Furthermore, a Task-Aligned Assigner replaces legacy IoU-based label allocation strategies, improving the precision of positive/negative sample selection through task-aware alignment.

#### 2.2.2. P2 Feature Pyramid Layer Integration for Small-Object Detection

**Figure 3:**
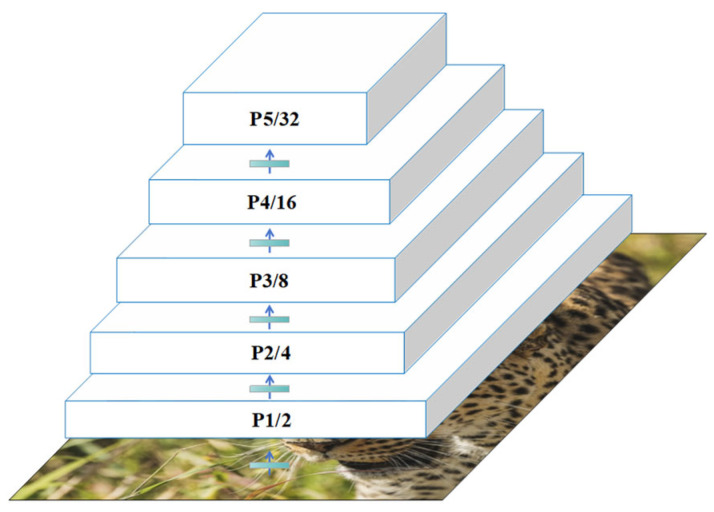
Illustration of Feature Pyramid Structure.

In the YOLOv8 architecture, the backbone network generates five-scale feature maps through five consecutive downsampling operations with a stride of 2, denoted as P1/2, P2/4, P3/8, P4/16, and P5/32, where the numerical subscripts indicate their downsampling ratios relative to the input image resolution (Figure 3). The coarsest feature map (P5/32) undergoes processing through the C2f module and Spatial Pyramid Pooling (SPP) to produce multi-scale fused features, which are subsequently propagated to the neck network for hierarchical feature aggregation.

In the neck network, the model employs a Feature Pyramid Network (FPN) [35] to process multi-scale feature maps (P3/8, P4/16, and P5/32), which correspond to small, medium, and large object detection requirements, respectively. However, the P3/8 layer (80 × 80 resolution), serving as the smallest detection layer, exhibits insufficient resolution for ultra-small targets (<32 × 32 pixels), leading to detail loss and degraded detection performance.

To address this limitation, the P2/4 layer (160 × 160 resolution) extracted from the backbone network was introduced into the original FPN structure of the neck network. This layer is designated as the dedicated feature hierarchy for ultra-small objects. By incorporating additional upsampling and downsampling operations, the P2/4 layer’s high-resolution shallow features are integrated into the FPN, mitigating detail loss caused by inadequate resolution. This refinement enriches feature propagation and fusion with spatially rich information, enabling coverage of a broader target scale spectrum. Concurrently, a P2/4 feature fusion branch is added to the detection head to specifically optimize ultra-small object detection, thereby extending the model’s capability to handle a wider range of target sizes.

#### 2.2.3. BiFPN_Concat-Driven Feature Fusion for Complex Scene Robustness

**Figure 4:**
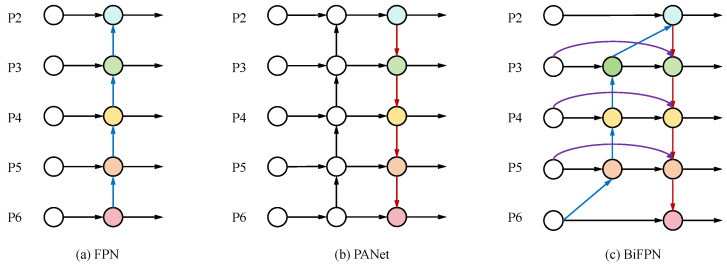
Comparison of Various Feature Pyramid Network Architectures: (**a**) FPN; (**b**) PAN-FPN; (**c**) BiFPN.

The Path Aggregation Feature Pyramid Network (PAN-FPN) in YOLOv8 achieves multi-scale feature fusion through bidirectional top-down and bottom-up pathways, where the red arrows in Figure 4 indicate the top-down information flow across feature levels. However, its fusion process occurs only once without adaptively optimizing the contribution of different features through weighted mechanisms, thereby limiting the depth and efficiency of feature interactions, as illustrated in Figure 4a,b. In contrast, the Bidirectional Feature Pyramid Network (BiFPN) [36], shown in Figure 4c, enhances traditional feature pyramid architectures for visual feature fusion by establishing multiple bidirectional connections across different scale features. In this figure, the red arrows indicate the top-down information flow, the blue arrows represent the bottom-up information flow, and the purple arrows denote lateral connections across different feature levels. This architecture enables iterative information exchange between multi-scale features while incorporating learnable weight parameters for each input feature, allowing the network to autonomously determine the relative importance of distinct feature representations.

To further enhance the model’s capability in detecting multi-scale features, this study optimizes the connection layers by introducing an improved BiFPN-based module named BiFPN_Concat, which includes two variants: BiFPN_Concat2 (dual-channel weighting) and BiFPN_Concat3 (triple-channel weighting). The core functionality of this module lies in adaptively fusing multiple input feature maps through learnable weighted parameters before concatenating them along specified dimensions. During initialization, the fusion dimensions and initial weights are predefined, while the forward propagation process dynamically normalizes these weights to achieve adaptive feature integration. BiFPN_Concat optimizes feature fusion through the following characteristics: enabling weighted aggregation of heterogeneous features, maintaining computational efficiency through dimension-aware concatenation, and enhancing contextual awareness by iteratively refining cross-scale dependencies through bidirectional pathways.

The BiFPN_Concat module achieves enhanced feature fusion through three principal mechanisms:

1. Iterative bidirectional fusion, where repeated bidirectional fusion blocks enable cyclic propagation of multi-scale features across the network, thereby amplifying feature representation capacity and fostering comprehensive cross-scale interactions through progressive refinement.
2. Adaptive weighted feature fusion, which introduces learnable weight parameters to dynamically optimize the proportional contributions of heterogeneous-scale feature maps. This weighting mechanism empowers task-aware prioritization of critical features by autonomously adjusting their importance during training.
3. Cross-layer connectivity, facilitating direct interactions between non-adjacent hierarchical layers during concatenation to ensure global feature consistency and unified contextual encoding across spatial and semantic hierarchies.

For dual-channel fusion, given two input feature maps *x* = [*x*_0_, *x*_1_], the process first applies learnable weights to perform weighted fusion and normalizes the corresponding weights into *weight_i_* (as defined in Equation (1)), where a small constant ϵ is introduced to prevent division by zero. Subsequently, each feature map is multiplied by its corresponding normalized weight to generate *w*_0_ *weight*_0_ and *w*_1_ *weight*_1_. Finally, the weighted feature maps are concatenated along a specified dimension (default *dim* = 1) to complete multi-scale feature fusion and produce the output, as formalized in Equation (2).

(1) $$weight_{i}=\frac{w_{i}}{w_{0}+w_{1}+\in }(i=0,1;\;\in =0.01)$$

(2) $$Output=Concat([w_{0}\;weight_{0},\;w_{1}\;weight_{1}],\;dim=1)$$

The triple-channel fusion process follows a methodology analogous to the dual-channel approach, where input feature maps undergo weighting, normalization, and concatenation along specified dimensions to achieve multi-scale feature integration. The primary distinction lies in expanding the input feature maps from two to three, necessitating normalization and weighted fusion of three learnable parameters *weight*_0_, *weight*_1_, *weight*_2_. This extension enhances the module’s capacity to capture hierarchical dependencies across broader feature hierarchies while maintaining computational efficiency through dimension-aware aggregation.

Integration of the BiFPN_Concat module into YOLOv8 involves replacing the original concatenation layers with BiFPN_Concat2 and BiFPN_Concat3. Specifically, during the top-down pathway, upsampled features are fused with shallow backbone features via BiFPN_Concat2 for dual-channel weighted fusion. In the bottom-up pathway, downsampled features are combined with both backbone and neck features using BiFPN_Concat3 for triple-channel weighted fusion. The improved module employs learnable parameters *self.w* to dynamically adjust feature weights, ensuring enhanced emphasis on semantically critical features during fusion.

#### 2.2.4. Multi-Head Self-Attention (MHSA) Mechanism

**Figure 5:**
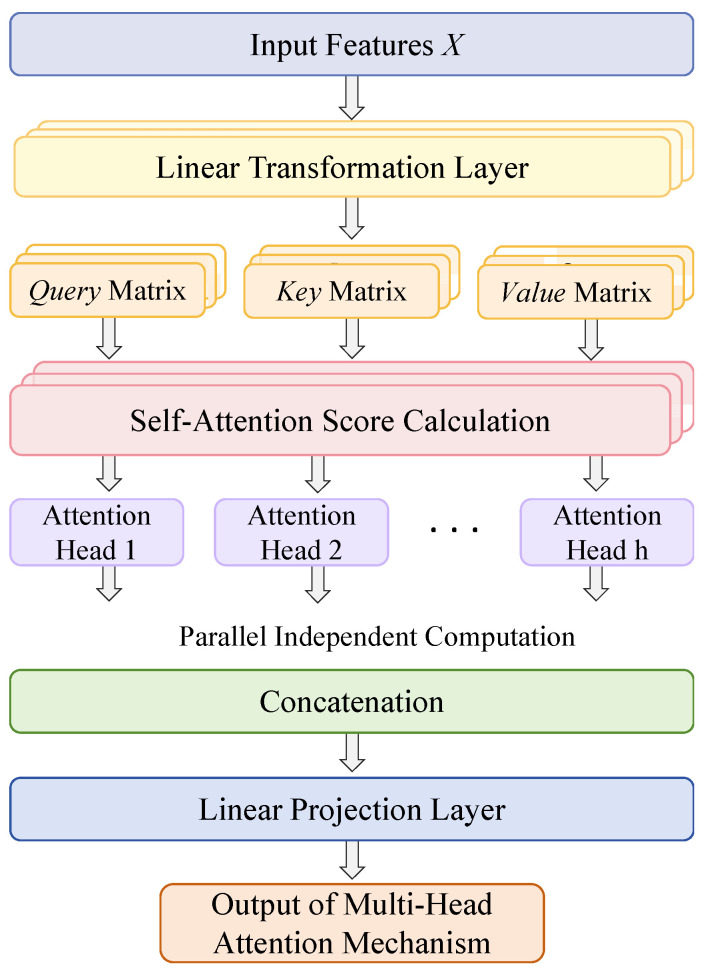
Illustration of MHSA Structure.

The core idea of Multi-Head Self-Attention (MHSA) lies in computing attention across multiple heads in parallel, enabling the model to capture rich contextual information and diverse feature representations (Figure 5). MHSA is an extension of the self-attention mechanism, which allows each element in the input sequence to attend to all other elements and dynamically compute a weighted average as its output. This approach enhances the model’s ability to understand and process image features more effectively.

The core of this process lies in the computation and weighting of the Query (*Q*), Key (*K*), and Value (*V*) matrices. The query represents the current focus of attention and is used to match positional information across the input; the key encodes identifiers of different positions within the input data and is used to compute relevance with the query; the value contains the actual content at each position and is aggregated via attention-weighted summation to produce the output.

The computation of Multi-Head Self-Attention (MHSA) begins with the input feature matrix $X\in R^{N\times d}$, where *N* denotes the input sequence length or the number of spatial positions after flattening, and *d* represents the feature dimension. The matrix *X* is transformed into the Query, Key, and Value matrices through three learnable linear projections (Equation (3)). The weight matrices *W_q_*, *W_k_*, and *W_v_* are trainable parameters that project *X* into the query, key, and value spaces, respectively (as shown in Equation (4)), where *R* denotes the set of real numbers.

(3) $$Q=W_{q}X,\;\;K=W_{k}X,\;\;V=W_{v}X$$

(4) $$X\in R^{N\times d},\;\;W_{q}=R^{d\times d_{q}},\;\;W_{k}\in R^{d\times d_{k}},\;\;W_{v}=R^{d\times d_{v}}$$

To implement the multi-head mechanism, the Query (*Q*), Key (*K*), and Value (*V*) matrices are split along the feature dimension into *h* separate heads. Each head has a dimensionality of *d*/*h*, allowing the model to compute attention in multiple subspaces in parallel, as shown in Equation (5). For the *i*-th head, the segmented matrices represent the corresponding input subspace, enabling each head to focus independently on different feature subspaces. Within each head, the scaled dot-product attention mechanism is applied to compute attention weights and generate the corresponding weighted output.

(5) $$Q^{i},K^{i},V^{i}\in R^{N\times (d/h)}\;\;i\in \{1,2,....,h\}$$

The computation of self-attention consists of three steps. First, the dot product between each row of the Query matrix *Q* and each column of the Key matrix *K* is calculated to produce a score matrix, which measures the similarity between *Q_i_* and *K_i_*. To mitigate the issue of vanishing gradients caused by large dot-product values in high-dimensional spaces, the scores are scaled by the square root of the dimensionality *d_k_* of each head. This process is described in Equation (6), where *d_k_* denotes the dimensionality of each attention head.

(6) $$AttentionScores^{i}=\frac{Q^{i}{\left(K^{i}\right)}^{T}}{\sqrt{d_{k}}}$$

Next, the attention scores are normalized using the softmax function to convert the raw scores into attention weights, as shown in Equation (7).

(7) $$AttentionWeights^{i}=Softmax(AttentionScores^{i})=Softmax(\frac{Q^{i}{\left(K^{i}\right)}^{T}}{\sqrt{d_{k}}})$$

Finally, the attention weights are used to compute a weighted sum of *V_i_*, producing the output of the *i*-th head. This output represents a feature embedding enriched with global contextual information, as shown in Equation (8).

(8) $$Head^{i}=AttentionWeights^{i}\cdot V^{i}$$

Therefore, the core of the self-attention mechanism lies in computing attention weights using *Q* and *K*, and applying these weights to *V* to generate the output. After computing the attention outputs for all heads, the results are concatenated along the feature dimension to form a matrix of shape *N × d*, as shown in Equation (9). A trainable weight matrix is then used to project the concatenated results to the final output dimension, which constitutes the computation of the multi-head mechanism, as expressed in Equation (10). Here, $W_{o}\in R^{d\times d}$, and $MHSA(X)\in R^{N\times d}$, maintaining consistency with the input dimensionality.

(9) $$MultiHeadOutput=Concat(Head^{1},Head^{2},\ldots ,Head^{h})$$

(10) $$MHSA(X)=Concat(Head^{1},Head^{2},Head^{h})W_{o}$$

MHSA captures global contextual information from the input feature maps through self-attention computation, thereby overcoming the limitations of local receptive fields inherent in traditional convolutional neural networks and enhancing the model’s holistic understanding of the image. Moreover, MHSA is capable of establishing long-range dependencies by linking distant pixels across the feature map, which is particularly beneficial for handling scenarios involving occluded or partially visible objects. The multi-head structure further enriches feature representation diversity by extracting information from multiple subspaces, enabling the model to better accommodate multi-scale and multi-class targets, thereby improving adaptability and representational capacity.

However, introducing MHSA in the early stages of the backbone network can significantly increase computational overhead and potentially interfere with the local feature extraction of subsequent convolutional layers. Therefore, MHSA is placed at the final layer of the backbone network, where the features—having undergone spatial pyramid fusion—contain rich semantic information at a relatively low resolution. This positioning allows the global feature capturing ability of MHSA to enhance semantic representation while maintaining computational efficiency.

### 2.3. Evaluation Indicators

**Figure 6:**
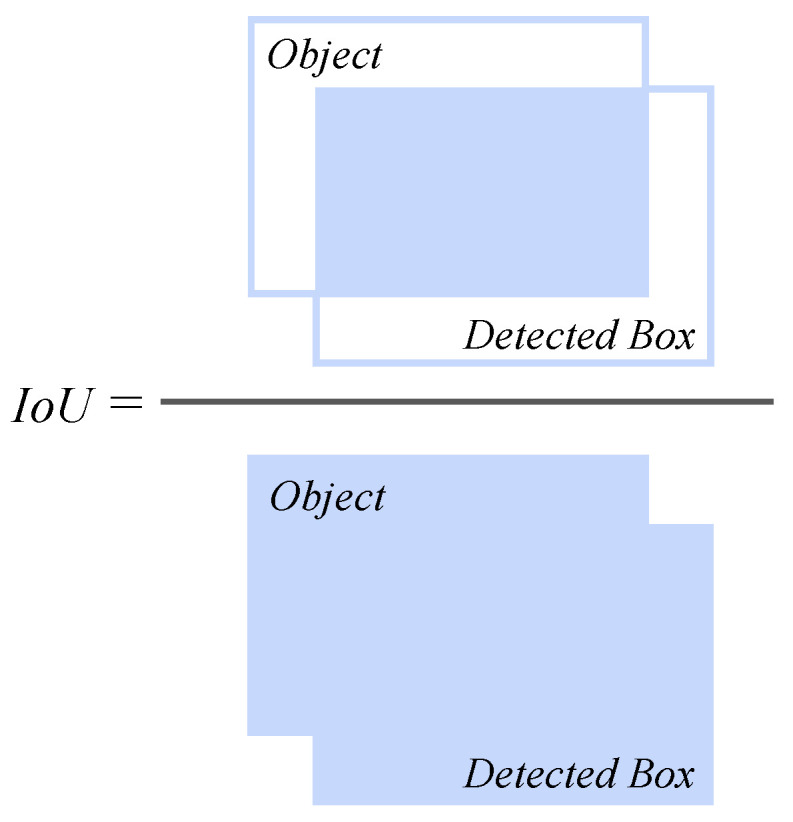
IoU calculation diagram.

Intersection over Union (IoU) measures the overlap between the predicted bounding box and the ground-truth bounding box, and is defined as the area of their intersection divided by the area of their union (Figure 6). It serves as a core metric for assessing object detection performance (Equation (11)). Confidence score reflects the model’s certainty regarding its predictions, calculated as the product of the probability of object presence P(Object) and the IoU value, ranging from 0 to 1. YOLOv8 retains predictions with high confidence scores by applying a predefined threshold (e.g., 0.5). Precision P indicates the proportion of true positive detections among all positive predictions, reflecting the accuracy of the detection results (Equation (12)). True Positives (TP) refer to correctly detected positive instances, while False Positives (FP) denote instances incorrectly predicted as positive, representing false detections. Recall R measures the proportion of true positive detections among all actual positives, reflecting the completeness of the detection (Equation (13)). False Negatives (FN) indicate instances where the model fails to detect actual positive cases, representing missed detections. The F1-Score is the harmonic mean of precision and recall, providing a balanced measure of both accuracy and completeness (Equation (14)). Mean Average Precision (mAP) is used to evaluate multi-class detection performance and is calculated as the mean of the Average Precision (AP) across all classes. In particular, mAP50 (IoU = 0.5) is the most critical metric in object detection experiments, representing the model’s detection capability at a 50% IoU threshold (Equation (15)). In the equation, APi denotes the average precision for the *i*-th class, and N is the total number of classes.

(11) $$IoU=\frac{Intersection\;Area}{Union\;Area}$$

(12) $$Precision=\frac{TP}{TP+FP}=\frac{TP}{All\;Detections}$$

(13) $$Recall=\frac{TP}{TP+FN}=\frac{TP}{All\;Ground\;Truths}$$

(14) $$F1-Score=2\times \frac{P\times R}{P+R}$$

(15) $$mAP50=\frac{1}{N}\sum_{i=1}^{N}AP_{i}(IoU=0.5)$$

## 3. Experimental Evaluation

### 3.1. Experimental Environment

The experimental environment is configured as follows: the hardware consists of an Intel(R) Xeon(R) Platinum 8255C CPU and a single NVIDIA RTX 2080 Ti GPU with 11 GB of memory and driver version 550.90.07. The software environment is based on the Ubuntu 20.04.3 LTS operating system, with Python 3.8 as the programming language and CUDA 11.3 for GPU acceleration. All experiments were conducted using the PyTorch 1.10.0 deep learning framework within a Python 3.8 virtual environment. The version of YOLOv8 used in the experiments is 8.0.135.

In YOLOv8 training, the patience parameter governs the early stopping strategy by specifying the number of additional training epochs allowed without improvement in the validation loss. The maximum number of training epochs was set to 5000—well beyond what is typically required for convergence—and the patience was set to 50, meaning that training would stop if the validation loss did not improve for 50 consecutive epochs. This setup ensures the model converges sufficiently and allows the proposed improvements to take full effect. The batch size was set to 16 and the number of data loading workers to 8, with all other hyperparameters kept at the YOLOv8 default settings.

### 3.2. Experimental Results and Analysis

#### 3.2.1. Ablation Experiment

The ablation study is designed to comprehensively evaluate the individual contributions of the three proposed improvements—namely, the addition of the P2 ultra-small object detection layer, the integration of BiFPN for optimized feature fusion, and the application of the MHSA multi-head attention mechanism at the final layer of the backbone network. By introducing each module individually and in combination, the study quantitatively assesses their respective impacts on overall model performance, focusing on improvements in detection accuracy, completeness, and multi-scale object detection capability.

The original YOLOv8 model was used as the baseline, and all experiments were conducted on the custom-built wildlife dataset. To ensure the comparability of results, all models were trained and evaluated under identical hardware conditions and training configurations. The primary evaluation metric was mAP50, supplemented by precision (P), recall (R), F1-Score, and mAP50-95 as secondary indicators. Detailed ablation results are presented in Table 2.

The results of the ablation study demonstrate that each of the proposed modules, as well as their various combinations, significantly enhance the performance of the YOLOv8 model. Moreover, the modules exhibit complementary effects in improving wildlife object detection capabilities. When assessed individually, each module contributes positively to the model’s performance: the P2 layer enhances small object detection, leading to a 0.006 increase in mAP50; BiFPN improves multi-scale feature fusion, resulting in a 0.009 increase in mAP50; and MHSA enhances adaptability in complex scenarios, yielding a 0.014 increase in mAP50.

Pairwise combinations further reveal synergistic effects among the modules. For instance, the integration of P2 and BiFPN improves multi-object detection, while the combination of P2 and MHSA enhances performance in detecting small and occluded objects. These configurations lead to more substantial improvements in mAP50 compared to individual enhancements. The full integration of all three modules in YOLO-WildASM achieves the most significant performance gains, particularly in mAP50 and F1-Score, confirming the effectiveness and superiority of the proposed strategies for wildlife detection on the custom dataset within the YOLOv8 framework.

#### 3.2.2. Comparative Experiment

A series of comparative experiments were conducted to validate the effectiveness of the proposed YOLO-WildASM model and ensure its superior performance in wildlife detection tasks. Considering that differences in model architecture, training strategies, and parameter scales may introduce uncontrolled variables and compromise result comparability, this study selected state-of-the-art models within the YOLO series for comparison. Furthermore, as model parameter size directly affects both detection performance and computational efficiency, all comparison models were chosen to be within the same parameter scale as YOLOv8 to eliminate performance bias resulting from differences in model complexity.

In the comparative experiments, YOLOv8 through YOLOv11 were implemented using CUDA version 11.3 and the PyTorch 1.10.0 deep learning framework. In contrast, YOLOv12 relies on new features introduced in PyTorch 2.0 and later, and was therefore evaluated using CUDA version 11.8 and PyTorch 2.4.1. The results of the comparative experiments are presented in Table 3.

The results of the comparative experiments demonstrate that the proposed YOLO-WildASM model outperforms the more recent YOLOv8-based models—YOLOv9t, YOLOv10n, and YOLOv11n—across all key evaluation metrics, including precision (P), recall (R), F1-Score, mAP50, and mAP50–95. Furthermore, YOLO-WildASM also exceeds the performance of the latest released YOLOv12n, achieving a notable advantage of 0.019 in mAP50. In addition, the recall and F1-Scores of YOLO-WildASM reach 0.888 and 0.905, respectively, both higher than those of other models within the same parameter scale, indicating superior detection capability in complex background scenarios.

It is particularly noteworthy that YOLO-WildASM even surpasses YOLOv12s—a model with a larger parameter size—in overall performance. Specifically, YOLO-WildASM achieves a 0.004 higher mAP50, along with slightly better recall and F1-Score. Although YOLOv12s shows a marginal advantage in mAP50–95, with YOLO-WildASM trailing by 0.006, the higher mAP50 value of YOLO-WildASM indicates stronger accuracy in detection tasks under the IoU threshold of 0.5. This performance advantage validates the effectiveness of the proposed improvements, enabling YOLO-WildASM to achieve superior detection capability without increasing model complexity.

**Figure 7:**
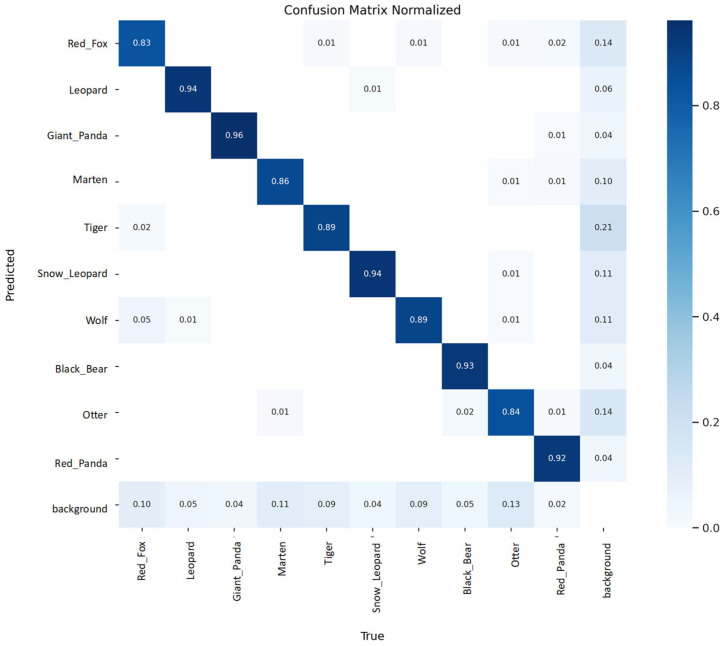
Normalized confusion matrix of YOLO-WildASM on the test set.

Figure 7 presents the normalized confusion matrices of the baseline YOLOv8n model and the proposed YOLO-WildASM on the test set, providing a detailed per-class evaluation of detection performance. Overall, YOLO-WildASM demonstrates improved classification accuracy and reduced inter-class confusion across most wildlife categories. For instance, the recognition accuracy of Red Panda increases from 0.83 to 0.94, Snow Leopard from 0.86 to 0.94, and Black Bear from 0.92 to 0.93, indicating enhanced discriminative capability in visually similar taxa. Similarly, improvements are observed for Martens and Otters, two classes highly susceptible to misclassification as background, where the refined feature representation introduced by the P2 detection branch and BiFPN fusion reduces false assignments and strengthens robustness against background interference.

Moreover, the proposed model effectively mitigates inter-class ambiguities in species pairs with overlapping visual traits, such as Tiger versus Snow Leopard and Wolf versus canid-like categories, as reflected by the lower off-diagonal values in YOLO-WildASM. This suggests that the MHSA module contributes substantially to capturing global contextual dependencies and suppressing spurious correlations between semantically related categories.

It should also be noted, however, that the background class exhibits heterogeneous trends. While background misclassification decreases for certain categories (e.g., Black Bear, from 0.06 to 0.04), it increases slightly for others, such as Red Fox (0.10 to 0.14) and Otter (0.13 to 0.14). This indicates that, although high-resolution shallow features introduced by the P2 branch enhance small-object detection, they may simultaneously increase sensitivity to background noise in low-contrast or fine-grained scenarios.

In summary, the confusion matrix analysis substantiates the quantitative improvements reported in Table 3. YOLO-WildASM exhibits superior per-class detection accuracy, particularly for small, occluded, and visually similar species, while maintaining competitive robustness in background suppression. These findings further validate the effectiveness of the ASM mechanism in improving both inter-class discrimination and overall detection reliability.

#### 3.2.3. Model Complexity Comparison

Beyond accuracy metrics, it is equally important to systematically evaluate the computational complexity and inference efficiency of a model, particularly in application scenarios where hardware resources are limited and real-time performance is critical. Therefore, we further conducted a quantitative analysis of the structural complexity and runtime efficiency of the proposed YOLO-WildASM compared with the YOLOv8n baseline. The results are summarized in Table 4.

As shown in the table, YOLO-WildASM, which is based on YOLOv8n and integrates a P2 small-object detection layer, BiFPN_Concat, and the MHSA attention mechanism, increases network depth from 168 to 212 layers. These architectural enhancements lead to an increase in the number of parameters from 3.0 M to 3.2 M, model size from 6.2 MB to 6.8 MB, and computational complexity from 8.1 GFLOPs to 12.5 GFLOPs. These increments are consistent with the introduction of the ASM modules, reflecting the enhanced representational capability of the network.

In terms of inference efficiency, YOLO-WildASM achieves an average latency of 3.3 ms and a throughput of 303 FPS, compared with 2.4 ms and 417 FPS for YOLOv8n. Although the speed decreases slightly due to additional computational overhead, YOLO-WildASM still maintains over 300 FPS, far exceeding the commonly accepted threshold of 30 FPS for real-time detection tasks.

YOLO-WildASM achieves a well-balanced trade-off between accuracy and efficiency. The modest increases in model size and computational cost are fully offset by the substantial improvements in detection accuracy. This confirms that the proposed enhancements not only improve detection performance but also preserve high efficiency, ensuring strong applicability in real-world deployment scenarios. This demonstrates that YOLO-WildASM retains strong real-time performance, making it well-suited for deployment on edge devices in wildlife conservation applications.

#### 3.2.4. Extended Comparative Experiment

To further validate the generalizability and robustness of the proposed ASM mechanism, we extended the evaluation to the more advanced YOLOv11n architecture. Compared with YOLOv8n, YOLOv11n incorporates several structural refinements in backbone and feature aggregation, making it a stronger and more competitive baseline within the YOLO family.

In implementation, the three improvements originally designed for YOLOv8n were fully transferred to YOLOv11n. First, the P2/4 layer was preserved in the backbone and integrated into the BiFPN structure of the neck through additional upsampling and downsampling paths, with a new P2 detection branch added in the head to strengthen ultra-small object detection. Second, the standard Concat operations in the detection head were replaced with the improved BiFPN_Concat2 and BiFPN_Concat3 modules, enabling adaptive weighted fusion of cross-scale features and improving contextual consistency. Finally, given that the original YOLOv11n already employed a C2PSA block at the P5 stage, we further introduced an MHSA module after C2PSA to enhance global contextual modeling and long-range dependency capture. The resulting YOLOv11n-ASM thus incorporates P2–P5 four-scale detection heads, BiFPN-based adaptive feature fusion, and deep-stage MHSA enhancement as a comprehensive design.

The experiments were conducted on the self-constructed dataset developed in this study to ensure alignment with practical conservation scenarios. As shown in Table 5, YOLOv11n-ASM achieves consistent improvements over the baseline YOLOv11n across multiple metrics: precision increases from 0.901 to 0.907, recall from 0.865 to 0.872, F1-Score from 0.883 to 0.903, mAP50 from 0.922 to 0.925, and mAP50-95 from 0.757 to 0.759. For comparison, the table also reports the results of YOLO-WildASM, which integrates the ASM mechanism into YOLOv8n. This model achieves the highest overall performance, with a precision of 0.922, a recall of 0.888, an F1-Score of 0.905, and mAP50 reaching 0.941.

The results show that the proposed ASM mechanism not only provides substantial performance gains on YOLOv8n but can also be successfully transferred to YOLOv11n while maintaining consistent improvements. This validates the adaptability and robustness of the method and confirms its value as a generalizable enhancement strategy for object detection across different architectures and application scenarios.

#### 3.2.5. Cross-Dataset Generalization Experiment

To verify whether the YOLO-WildASM model can maintain high performance across diverse data environments, this study designs a cross-dataset experiment. The experiment employs a publicly available dataset, African-Wildlife, provided by Ultralytics, which is entirely independent of the custom dataset used for training. This dataset comprises 1504 annotated images featuring four common animal species found in South African nature reserves: buffalo, elephant, rhinoceros, and zebra. The dataset is split into 1052 training images, 225 validation images, and 227 test images, with a ratio of 0.7:0.15:0.15. Given the differences in both species composition and imaging environments between this dataset and the custom dataset, it provides a suitable testing scenario for evaluating the generalization capability of the proposed model.

As shown in Table 6, the results demonstrate that YOLO-WildASM performs competitively on the African-Wildlife dataset across multiple evaluation metrics. Notably, it achieves mAP50 and mAP50–95 scores of 0.973 and 0.82, respectively. With the exception of a slightly lower mAP50–95 compared to YOLOv12s, YOLO-WildASM outperforms all other baseline models, including the larger-parameter YOLOv11s and YOLOv12s. This advantage highlights YOLO-WildASM’s strong generalization capability in wildlife recognition, indicating its suitability for detecting various wildlife species and maintaining stable performance in diverse field environments.

Beyond the mAP metrics, YOLO-WildASM achieves a precision (P) of 0.950, which is slightly lower than YOLOv12n’s 0.953 but still higher than all other evaluated models. This marginal difference suggests that YOLO-WildASM closely matches the latest YOLOv12n in terms of reducing false positives, thereby maintaining high detection accuracy. The recall (R) of YOLO-WildASM reaches 0.916, which is slightly lower than that of YOLOv11s, but still exceeds that of the remaining models. Moreover, its F1-Score is nearly equivalent to that of YOLOv11s, indicating a well-balanced performance between precision and recall. This balance reflects strong object coverage, allowing the model to identify targets more comprehensively while maintaining a low miss rate.

The outstanding performance of YOLO-WildASM in the cross-dataset generalization experiment validates the effectiveness of its proposed improvement strategies and demonstrates strong generalization capability. These results demonstrate the potential of YOLO-WildASM as a general-purpose model for wildlife detection. The model is well-suited for broader application in the identification and monitoring of diverse species.

## 4. Discussion

### 4.1. Estimation Accuracy and Portability of YOLO-WildASM

This study aims to address key challenges in wildlife object detection, including complex backgrounds, small object sizes, target occlusions, and the coexistence of multiple objects. Based on the YOLOv8 architecture, an improved model, YOLO-WildASM, is proposed and optimized for ten representative wildlife species with distinct ecological and visual characteristics. The model incorporates three core enhancements: a P2 ultra-small object detection layer, a BiFPN feature fusion mechanism, and a Multi-Head Self-Attention (MHSA) module. These enhancements significantly improve the model’s capability to handle small targets, occluded objects, multi-object scenarios, and visually complex environments.

Experimental results demonstrate that YOLO-WildASM outperforms YOLOv8 and its subsequent versions across several key performance metrics, confirming the effectiveness of the proposed improvements. Specifically, the P2 layer enhances small object detection by preserving high-resolution features; BiFPN improves feature integration through repeated bidirectional and weighted fusion, thereby enhancing robustness and accuracy in complex scenes; and MHSA captures global contextual information, overcoming the limitations of local receptive fields in conventional convolutions and improving the model’s semantic understanding of images.

Ablation studies further validate the individual contributions and synergistic effects of the proposed modules. For instance, the P2 layer significantly enhances small object detection, while the combination of BiFPN and MHSA contributes to improved overall robustness. Cross-dataset generalization experiments indicate that YOLO-WildASM maintains strong performance across datasets with varying characteristics, demonstrating its adaptability and potential for deployment in diverse wildlife detection scenarios. In addition, model complexity analysis shows that YOLO-WildASM achieves a balanced trade-off between accuracy and efficiency, sustaining real-time inference above 300 FPS despite a slight increase in parameters and FLOPs. Extended experiments further confirm that the ASM mechanism can be successfully transferred to YOLOv11n, where it consistently improves precision, recall, and mAP, highlighting the robustness and portability of the proposed approach.

**Figure 8:**
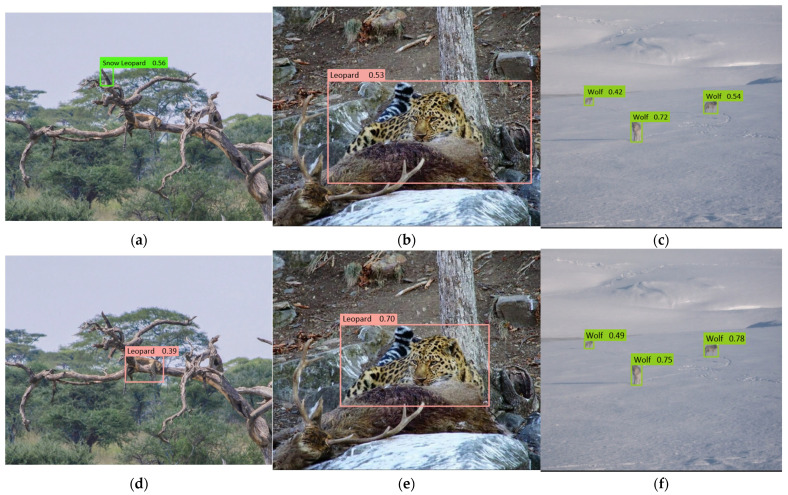
Model optimization effect comparison. (**a**) False positives by baseline model; (**b**) Localization errors by baseline model; (**c**) Low confidence predictions by baseline model; (**d**) False positive suppression by enhanced model; (**e**) Precise localization by enhanced model; (**f**) High-confidence predictions by enhanced model.

**Figure 9:**
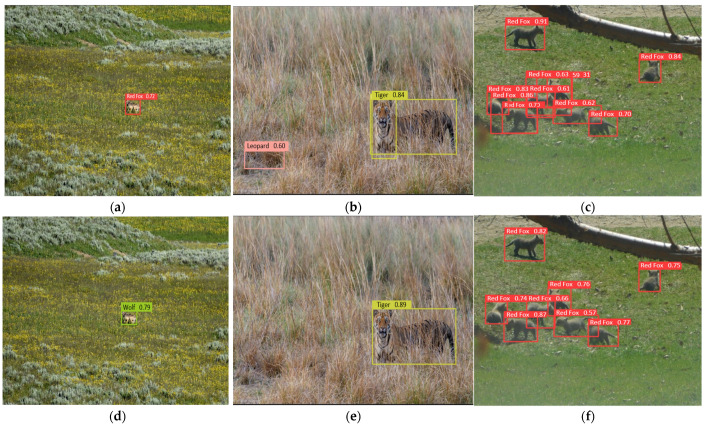
Model optimization effect comparison: (**a**) Low confidence for small objects by baseline model; (**b**) Localization failure under occlusion by baseline model; (**c**) Target confusion in multi-object scenes by baseline model; (**d**) Accurate small object detection by enhanced model; (**e**) Robust occlusion handling by enhanced model; (**f**) Clear multi-target differentiation by enhanced model.

Figure 8 and Figure 9 illustrate the detection results before and after model enhancement. The improved model effectively addresses detection challenges in complex backgrounds, such as false positives, inaccurate localization, and low confidence predictions. It also shows superior performance in scenarios involving small objects, occlusions, and multiple targets. In natural environments such as grasslands and forested areas, YOLO-WildASM significantly reduces errors, including misclassification, redundant anchors, and missed detections, thereby achieving more accurate and reliable object localization and recognition.

### 4.2. Limitations of Modeling Methods

The YOLO-WildASM model is intentionally designed to function independently of geographic information—thereby ensuring generalizability in scenarios where such data is unavailable—this design choice may compromise detection efficiency in specific contexts. For example, species such as the giant panda and snow leopard exhibit highly distinct geographic distributions, and incorporating location priors could significantly reduce the search space and improve identification accuracy. With regard to architectural adaptability, the proposed improvements were originally developed on the YOLOv8 backbone and have been successfully transferred to the more advanced YOLOv11n architecture, where consistent gains in precision, recall, and mAP were achieved. This demonstrates that the ASM mechanism is not confined to a single generation of YOLO but can be effectively generalized to newer backbones. In addition, we explored extending the proposed ASM mechanism to the latest YOLOv12 framework to evaluate its applicability within more advanced architectures. However, this attempt revealed integration challenges primarily attributable to the unique architectural characteristics of YOLOv12. Compared with YOLOv8 and YOLOv11, YOLOv12 introduces substantial design differences, particularly stricter interface definitions governing feature extraction and attention integration. These architectural specifications led to a mismatch with the more flexible mapping process employed in the ASM mechanism, thereby restricting seamless integration in the current version.

We fully acknowledge this limitation and consider it an important direction for future work. Potential improvements include introducing dimension-adjustment modules during the feature mapping stage to enhance compatibility while maintaining the stability of YOLOv12, as well as designing lightweight adaptation layers within the ASM mechanism to increase its flexibility and portability. With such optimizations, the ASM mechanism holds strong potential not only for effective integration into YOLOv12 but also for broader deployment in subsequent generations of YOLO architectures.

In addition, for mobile or edge deployment, incorporating a location-based species probability classification module could be beneficial. By leveraging prior knowledge of species distribution, such a module could prioritize the detection of high-probability species, thereby improving both accuracy and inference speed. Expanding the training dataset to include a wider range of wildlife species would also enhance the model’s generalizability and practical utility. These improvements could provide more efficient technical solutions for applications in wildlife conservation and population monitoring.

In summary, YOLO-WildASM significantly enhances wildlife object detection performance through targeted architectural improvements, making important contributions to both theoretical research and real-world applications. Future optimizations will aim to further strengthen its adaptability and usability in complex environments, offering robust technological support for global wildlife conservation efforts.

## 5. Conclusions

This study addresses practical challenges in wildlife detection and recognition, particularly the limited detection accuracy observed in field monitoring scenarios due to factors such as complex backgrounds, small object sizes, multiple coexisting targets, and occlusions. To tackle these issues, we propose YOLO-WildASM, an improved object detection model based on the YOLOv8 framework. By introducing a P2 ultra-small object detection layer, a BiFPN feature fusion module, and a Multi-Head Self-Attention (MHSA) mechanism, the model demonstrates significantly enhanced performance in small object detection, multi-object scenarios, and complex environmental conditions.

Building upon a custom-constructed dataset representing ten ecologically and visually distinct wildlife species, we conducted comprehensive comparative experiments. The results show that YOLO-WildASM consistently outperforms YOLOv8 and its subsequent variants across all key evaluation metrics. Specifically, precision increased by 0.002 to 0.920; recall improved by 0.055 to 0.880; the F1-Score rose by 0.031 to 0.905; mAP50 increased by 0.028 to 0.941; and mAP50–95 improved by 0.041 to 0.777. These gains confirm the effectiveness and practicality of the proposed enhancements.

In addition, ablation experiments validated the individual contributions and synergistic effects of each module. Model complexity analysis confirmed that YOLO-WildASM achieves a balanced trade-off between accuracy and efficiency. Extended comparative experiments verified that the ASM mechanism can be successfully transferred to YOLOv11n. Cross-dataset generalization tests further demonstrated the model’s strong adaptability and transferability across diverse wildlife datasets and environments, underscoring its potential as a general-purpose wildlife detection model.

Overall, this study provides a targeted structural improvement to the YOLOv8 model while offering solid empirical evidence of its enhanced performance in real-world applications. YOLO-WildASM effectively addresses key limitations in conventional wildlife detection frameworks. Future research may explore lightweight deployment strategies, integration of geospatial priors to optimize detection, and migration to more advanced architectures such as YOLOv12. These directions will further broaden the applicability of YOLO-WildASM in wildlife conservation, intelligent ecological monitoring, and large-scale biodiversity data analysis.

## Figures and Tables

**Table 1:** Provenance of Dataset Images.

Animal Name	Chengdu Institute of Biology	iNaturalist	iStock	Landbridge Ecology Center	China Conservation and Research Center for the Giant Panda	Chengdu Research Base of Giant Panda Breeding	Total Images
Leopard		960					960
Giant Panda		13	338		524	34	909
Tiger		892	21				913
Snow Leopard		124	298	456			878
Red Fox	119	725		105			949
Yellow-throated Marten	106	709					815
Wolf	508	273		30			811
Black Bear	198	129	205				532
Otter	69	775					844
Red Panda	185	90			189	99	563

**Table 2:** Ablation Study Results.

Model	Precision (P)	Recall (R)	F1-Score	mAP50	mAP50-95
YOLOv8 (Baseline)	0.92	0.833	0.874	0.913	0.736
YOLOv8 + P2	0.922	0.848	0.884	0.919	0.741
YOLOv8 + BiFPN	0.911	0.852	0.881	0.922	0.742
YOLOv8 + MHSA	0.906	0.871	0.888	0.927	0.757
YOLOv8 + P2 + BiFPN	0.93	0.859	0.893	0.928	0.755
YOLOv8 + P2 + MHSA	0.91	0.872	0.891	0.933	0.767
YOLOv8 + BiFPN + MHSA	0.915	0.848	0.880	0.929	0.754
YOLO-WildASM	0.922	0.888	0.905	0.941	0.777

**Table 3:** Comparison Experiment Results.

Model	Precision (P)	Recall (R)	F1-Score	mAP50	mAP50-95
YOLOv8n	0.92	0.833	0.874	0.913	0.736
YOLOv9t	0.922	0.854	0.887	0.922	0.766
YOLOv10n	0.92	0.833	0.874	0.911	0.737
YOLOv11n	0.901	0.865	0.883	0.922	0.757
YOLOv12n	0.911	0.863	0.886	0.922	0.785
YOLOv12s	0.925	0.882	0.903	0.937	0.783
YOLO-WildASM	0.922	0.888	0.905	0.941	0.777

**Table 4:** Comparison of the proposed model and YOLOv8n.

Model	Layers	Parameters	GFLOPs	Size (MB)	Latency (ms)	FPS
YOLOv8n	168	3,007,598	8.1	6.2	2.4	417
YOLO-WildASM	212	3,205,767	12.5	6.8	3.3	303

**Table 5:** Performance of the proposed model and YOLOv11n, YOLOv11n-ASM.

Model	Precision (P)	Recall (R)	F1-Score	mAP50	mAP50-95
YOLOv11n	0.901	0.865	0.883	0.922	0.757
YOLOv11n-ASM	0.907	0.872	0.903	0.925	0.759
YOLO-WildASM (ours)	0.922	0.888	0.905	0.941	0.777

**Table 6:** Results of Cross-Dataset Generalization Experiment.

Model	Precision (P)	Recall (R)	F1-Score	mAP50	mAP50-95
YOLOv8n	0.927	0.888	0.907	0.95	0.8
YOLOv9t	0.946	0.888	0.916	0.95	0.799
YOLOv10n	0.942	0.876	0.908	0.954	0.808
YOLOv11n	0.937	0.89	0.913	0.946	0.807
YOLOv11s	0.939	0.93	0.934	0.967	0.807
YOLOv12n	0.953	0.895	0.923	0.964	0.8
YOLOv12s	0.932	0.904	0.918	0.966	0.821
YOLO-WildASM	0.95	0.916	0.933	0.973	0.82

## Data Availability

The original contributions presented in this study are included in the article. Further inquiries can be directed to the corresponding author.
